# Digital health behaviour change interventions targeting physical activity and diet in cancer survivors: a systematic review and meta-analysis

**DOI:** 10.1007/s11764-017-0632-1

**Published:** 2017-08-04

**Authors:** Anna L. Roberts, Abigail Fisher, Lee Smith, Malgorzata Heinrich, Henry W. W. Potts

**Affiliations:** 1 Department of Behavioural Science & Health, University College London, Gower Street, London, WC1E 6BT UK; 2 The Cambridge Centre for Sport and Exercise Sciences, Department of Life Sciences, Anglia Ruskin University, Cambridge, UK; 3 Institute of Health Informatics, University College London, London, UK

**Keywords:** Behaviour change, Digital interventions, Physical activity, Cancer survivors, Diet, Sedentary behaviour

## Abstract

**Purpose:**

The number of cancer survivors has risen substantially due to improvements in early diagnosis and treatment. Health behaviours such as physical activity (PA) and diet can reduce recurrence and mortality, and alleviate negative consequences of cancer and treatments. Digital behaviour change interventions (DBCIs) have the potential to reach large numbers of cancer survivors.

**Methods:**

We conducted a systematic review and meta-analyses of relevant studies identified by a search of Medline, EMBASE, PubMed and CINAHL. Studies which assessed a DBCI with measures of PA, diet and/or sedentary behaviour were included.

**Results:**

Fifteen studies were identified. Random effects meta-analyses showed significant improvements in moderate-vigorous PA (seven studies; mean difference (MD) = 41 min per week; 95% CI 12, 71) and body mass index (BMI)/weight (standardised mean difference (SMD) = −0.23; 95% CI −0.41, −0.05). There was a trend towards significance for reduced fatigue and no significant change in cancer-specific measures of quality of life (QoL). Narrative synthesis revealed mixed evidence for effects on diet, generic QoL measures and self-efficacy and no evidence of an effect on mental health. Two studies suggested improved sleep quality.

**Conclusions:**

DBCIs may improve PA and BMI among cancer survivors, and there is mixed evidence for diet. The number of included studies is small, and risk of bias and heterogeneity was high. Future research should address these limitations with large, high-quality RCTs, with objective measures of PA and sedentary time.

**Implications for cancer survivors:**

Digital technologies offer a promising approach to encourage health behaviour change among cancer survivors.

**Electronic supplementary material:**

The online version of this article (doi:10.1007/s11764-017-0632-1) contains supplementary material, which is available to authorized users.

## Introduction

Over 14 million people are diagnosed with cancer worldwide each year, and this is expected to rise to 22 million over the next two decades [1]. Improvements in early diagnosis and treatments mean that cancer survival is increasing. In 2012, globally there were 32 million people living beyond 5 years of diagnosis [2] and in the UK, half of people diagnosed with cancer will now survive for more than 10 years [3].

However, long-term negative consequences of cancer and treatment related side-effects are common and often debilitating. Prevalence of fatigue following a cancer diagnosis ranges from 59 to 100% depending on cancer type [4], and pain [5], sleep problems [6], physical side effects (e.g. lymphoedema) [7], weight gain [8], anxiety and depression [9, 10], fear of cancer recurrence [11] and impaired quality of life (QoL) [12] are all commonly reported. Macmillan Cancer Support, a UK cancer charity, estimates that more than 70% of cancer survivors in the UK (~1.8 million people) are also living with at least one other long-term comorbidity [13]. The most common comorbid conditions are hypertension, obesity, mental health problems and chronic heart disease [13]. The shared risk factors between cancer, obesity and cardiovascular disease (CVD) partially explain comorbidities [14]. However, there is also emerging evidence to suggest that cancer treatment can leave survivors at greater risk for developing these conditions (e.g. due to cardiovascular toxicity of cancer therapy [15]). The greater number and severity of comorbidities is linked to greater risk of death and cancer recurrence among cancer survivors [16]. There is now strong impetus to develop interventions that improve long-term outcomes for cancer survivors.

Health behaviours such as physical activity (PA), sedentary behaviour and diet are important in risk reduction and self-management of cancer, CVD and obesity. For example, a meta-analysis of 22 prospective cohort studies of 123,574 breast cancer survivors found that greater post-diagnosis PA participation reduced all-cause (hazard ratio [HR] = 0.52, 95% CI 0.43, 0.64) and breast cancer-specific mortality (HR = 0.59, 95% CI 0.45, 0.78), and breast cancer recurrence (HR = 0.79, 95% CI 0.63, 0.98) [17]. A meta-analysis of prospective studies of colorectal cancer survivors reported similar conclusions and showed that post-diagnosis PA reduced both all-cause (summary relative risk [RR] = 0.58; 95% CI 0.48, 0.70; *n* = 6 studies) and colorectal cancer-specific mortality (summary RR = 0.61; 95% CI 0.40, 0.92; *n* = 5 studies) [18]. The authors estimated that each 10 metabolic equivalent task (MET)-hour per week increase in post-diagnosis PA was associated with 24% (95% CI 11–36%) and 28% (95% CI 20–35%) decreased total mortality risk for breast and colorectal cancer survivors, respectively [18]. Mishra et al.’s meta-analysis of non-digital interventions focused on the effect of PA on health-related QoL (HRQoL) outcomes in cancer survivors (various types) and found that greater PA participation significantly improved overall HRQoL at up to 12 weeks of follow-up (11 studies, *n* = 826; standardised mean difference [SMD] = 0.48, 95% CI 0.16, 0.81) [19]. Individual meta-analyses of other cancer-relevant outcomes identified in this same Cochrane review also found that PA interventions improved emotional well-being/mental health and social functioning, and reduced anxiety, fatigue, pain and sleep disturbance [19]. Although limited to cross-sectional and prospective studies, there is some evidence that higher levels of sedentary time are associated with lower physical and role functioning domains of QoL, and greater reporting of comorbidities, disability and fatigue [20–22]. As a result of the growing evidence of the benefits of PA following a cancer diagnosis, cancer survivors are encouraged to avoid inactivity as far as possible and to meet the same PA guidelines as the rest of the adult population of at least 150 min of moderate-vigorous PA (MVPA) and two instances of strength/resistance-based exercises per week [23–25].

Diet may also influence outcomes following a cancer diagnosis. A meta-analysis of three studies (*n* = 9966) suggested that a low-fat diet post diagnosis can reduce breast cancer recurrence by 23% and all-cause mortality by 17% [26]. Another meta-analysis of four prospective cohort studies (*n* = 3675) found that high saturated fat intake increased breast cancer-specific mortality [27]. A meta-analysis of 56 observational studies in 1,784,404 cancer survivors (various types) showed greater adherence to a Mediterranean-style diet (largely based on vegetables, fruits, nuts, beans, cereal grains, olive oil and fish) was associated with lower all-cause cancer mortality for colorectal, breast, gastric, prostate, liver, head and neck, pancreatic and respiratory cancers [28]. Colorectal cancer survivors consuming a Western diet (high intake of meat, fat, refined grains and desserts) showed greater risk of recurrence and overall mortality compared to those with a prudent diet (high intake of fruits and vegetables, poultry and fish) in a prospective study of 1009 participants [29]. Similar findings have been shown in other prospective cohort studies of breast cancer survivors [30, 31]. Breast cancer survivors with better overall diet quality also reported lower levels of fatigue, independently of PA participation, at 41 months post diagnosis in a prospective cohort study [30].

Longitudinal studies have shown that obesity increases the risk of cancer recurrence among prostate [32], colorectal [33] and breast [34] cancer patients.

Despite the wealth of evidence, cancer survivors’ engagement with health behaviours and adherence to lifestyle guidelines for cancer survivors are remarkably poor [35, 36]. The English Longitudinal Study of Ageing demonstrated that the proportion of cancer survivors who engaged in self-reported MVPA at least once per week fell from 13% before their cancer diagnosis to 9% after their cancer diagnosis (compared to a fall of 16 to 15% in the group who did not receive a cancer diagnosis between data collection waves) [37]. Wang et al. found that cancer survivors were less likely to engage in self-reported PA (adjusted odds ratio = 0.79, 95% CI 0.67, 0.93) compared to those without a cancer diagnosis [38]. Furthermore, few cancer survivors meet the minimum recommended guidelines of 150 min of MVPA per week. A study of over 9000 survivors of six types of cancer found that adherence to PA recommendations varied from 30% (uterine cancer) to 47% (skin melanoma); however, this study did use self-reported PA measures [36]. While this study reported that 35% of breast cancer survivors were meeting guidelines, another study which used accelerometers to measure PA objectively found that this can be as low as 16% and those with highest levels of comorbidities were the least active [39]. Consequently, there is a need for evidence-based interventions that are easy to access, low-cost and which therefore have the feasibility to be rolled out to reach a large number of cancer survivors.

A move towards digital health behaviour change interventions (DBCIs) has been driven by widespread and rising use of the Internet, smartphones and mobile technology [40, 41]. The most recent Ofcom Communications Market report for the UK has shown that the proportion of adults going online using a mobile phone has risen from 20% in 2009 to 66% in 2016 and 71% of adults own a smartphone [41]. DBCIs use technologies such as text messaging, email, mobile applications (apps), video-conferencing (e.g. Skype), social media, websites and online patient portals increasing access to information, connecting patients with health services and as an approach to remote delivery of behaviour change interventions. DBCIs have been used in the promotion of medication adherence [42], management of long-term conditions [43–45], promoting smoking cessation [46] and promoting PA participation and dietary behaviours [47–50]. A recent systematic review of 224 studies reported that Internet and mobile interventions improved diet, PA, obesity, tobacco and alcohol use up to 1 year [51]. Among cancer survivors, a recent systematic review of 27 non-face-to-face intervention studies found telephone interventions as an effective approach to delivering PA and dietary interventions [52]. However, newer digital technologies should now be evaluated in this population as only three of the studies in that systematic review included used web-based methods to deliver the intervention [53–55]. No systematic review or meta-analysis has assessed the efficacy of DBCI interventions targeting PA, diet and/or sedentary behaviour among cancer survivors specifically.

Therefore, the primary objective of this study was to perform a systematic review and meta-analysis of health behaviour interventions using digital technologies in cancer survivors in order to assess their efficacy in promoting PA, reducing sedentary behaviour or improving dietary quality. Secondary aims were to explore any effects of DBCIs on BMI/weight, other cancer-relevant outcomes and the theoretical underpinning of included studies.

## Methods

### Search strategy

A systematic literature search was conducted from database inception to November 8, 2016, of the following databases: Medline, EMBASE, PsycINFO and CINAHL. Full details of the search strategy/terms used can be found in Online Resource 1. Broadly, the search strategy combined synonyms for PA, diet and/or sedentary behaviour; with types of DBCIs (e.g. website, mobile app, text messaging); and with words for cancer survivor(ship). Limits included peer-reviewed, English language articles in human subjects. Forward and backward citing of included studies and hand-searching of relevant journals were also conducted to identify relevant articles. The protocol was registered in the PROSPERO database (CRD42016026956). After piloting of the search strategy, no new or relevant articles from other databases specified in the protocol (Cochrane Library, Web of Science, ACM Digital Library, or IEEE Xplore) were identified so these databases were excluded for the final search. As specified in the protocol, the ProQuest database (grey literature) was searched; however, this resulted in >60,000 search results. Results were sorted by relevance, and the first 200 titles were reviewed. No additional, relevant papers which met criteria were identified throughout this process so grey literature was not included.

### Study selection

**Fig. 1:**
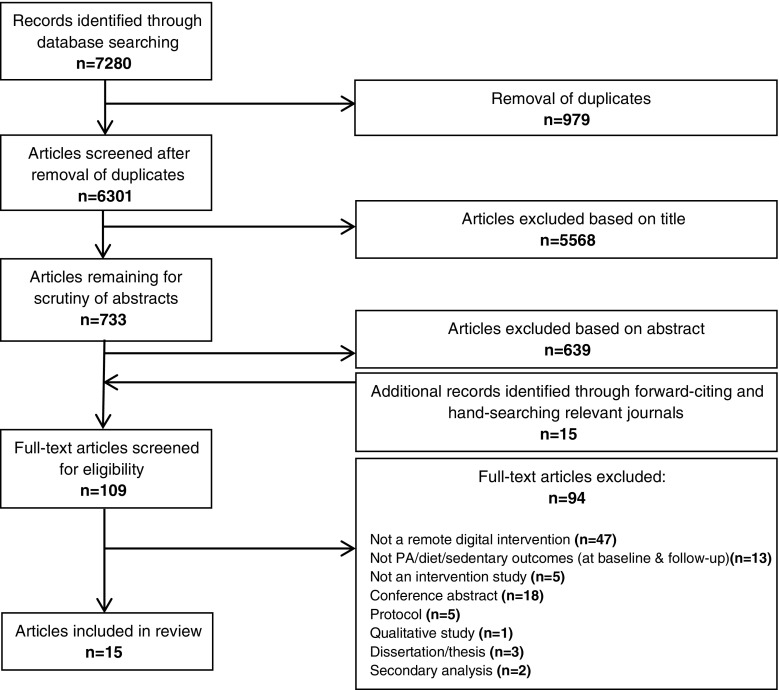
PRISMA flow diagram illustrating article selection strategy

Studies were selected in line with the search strategy shown in Fig. 1. Eligible studies included DBCIs delivered remotely and targeting at least one of the following health behaviours: PA, diet and/or sedentary behaviour in adults (≥18 years) who had a cancer diagnosis of any type. There were no restrictions on quantitative study designs, so both randomised and non-randomised controlled trials and one-arm pre-post comparison studies could be included. However, qualitative studies and protocols were excluded. Studies must have measured at least one of the target health behaviours (PA, diet and/or sedentary behaviour) at baseline and follow-up, but there were no limits on length of follow-up for inclusion.

### Data extraction and quality assessment

Two authors (AR and AF) independently reviewed 109 full-text articles screened for eligibility and extracted the data for included studies including author, country of study, study design, sample size, retention rate, population studied, age of participants, study duration, intervention type (i.e. type of DBCI), description of intervention content (including incorporated behaviour change techniques (BCTs)), approaches to measurement of engagement/adherence to the intervention, control group treatment and outcomes measured. Any discrepancies were resolved through discussion. Michie et al.’s BCT Taxonomy (v1) [56, 57] was used to code BCTs based on information provided in the included studies (and any supplementary material). The Cochrane Collaboration’s tool for assessing risk of bias was used to evaluate methodological quality of included studies [58], and Michie and Prestwich’s Theory Coding Scheme was used to evaluate the theoretical basis of the included studies [59].

### Statistical methods

Where possible, findings from both RCTs and one-arm pre-post studies were synthesised in random effects meta-analyses using Stata. Effect sizes for the intervention were calculated using the difference in final values between experimental and control groups in RCTs and the change in scores before and after the intervention in pre-post studies. It is not recommended to combine studies using a mixture of final values and change scores when using standardised mean differences (SMDs) across studies using different measurement units/tools to assess an outcome [58]. Therefore, outcomes using the same measurement unit were chosen wherever possible so non-standardised mean differences could be used and RCTs and pre-post studies could be combined in the meta-analyses [58]. Where this was not possible (i.e. fatigue outcomes), SMDs and their associated 95% CIs were calculated, and meta-analyses were conducted for the RCTs only (where the effect size reflects difference in final values between groups). As BMI is largely influenced by weight, the variability in reliability was judged to be similar for weight and BMI. Therefore, SMDs were used to pool the effect of BMI and weight across both the RCTs and pre-post studies reporting these outcomes. For PA outcomes, MVPA was chosen as the outcome measure of interest due to the American College of Sports Medicine’s recommendation that cancer survivors follow the PA guidelines for the general population of at least 150 min of at least moderate intensity PA per week [24]. Studies reporting MVPA duration in minutes were pooled in the meta-analysis, so studies with differences in final values and change scores could be used using mean differences. Studies that did not report moderate and vigorous PA separately or MVPA combined in minutes were not included in the meta-analysis of PA outcomes. For the studies that reported minutes of moderate and vigorous PA separately, a new combined MVPA variable was calculated. To combine the means for moderate and for vigorous PA, the following formula was used:

$$ {\overset{-}{x}}_{MVPA}={\overset{-}{x}}_{moderate\ PA}+{\overset{-}{x}}_{vigorous\ PA} $$

To combine the standard deviations for moderate and vigorous PA, the following formula was used:

$$ {\sigma}_{MVPA}=\sqrt{\left(\left({\sigma^2}_{moderate\ PA}\right)+\left({\sigma^2}_{vigorous\ PA}\right)\right)} $$

Publication bias was explored using funnel plots prepared in Stata. Due to the small number of included studies for each outcome, tests for funnel plot asymmetry (e.g. Egger’s regression test [60]) were not deemed appropriate and only visual inspection of funnel plots was conducted.

## Results

### Study selection

See Fig. 1 for the PRISMA flow diagram of the study selection process. The search strategy initially identified 7280 records, and 15 were included in the final review [53–55, 61–72].

See Table 1 for characteristics of included studies and Table 2 for characteristics of intervention types and outcomes. The majority of studies (12/15) were published between 2014 and 2016, with one study published in 2012 [54] and two in 2013 [53, 55]. Sample sizes ranged between 7 [64] and 462 [71]. Eight studies were RCTs [53–55, 61, 63, 67, 71, 72], and the remaining seven were pre-post comparison studies [62, 64–66, 68–70]. The studies used an average of eight BCTs (range 2–16). Self-monitoring of behaviour (*n* = 15), goal setting (behaviour) (*n* = 13), credible source (*n* = 13) and feedback on behaviour (*n* = 12) were the most frequently described BCTs. Short et al.’s study [72] was the only study which used a three-arm RCT design where all groups received the same intervention content, but the delivery schedule differed. As there was no true control, for the purposes of this review this study was treated as a pre-post. All 15 studies assessed the impact of the DBCIs on PA, five on diet [61, 67, 68, 70, 71], and no studies assessed the impact of DBCIs on sedentary behaviour.

**Table 1:** Characteristics of included studies

Author, year	Country	Study design	Sample size	Retention rate at follow-up	Women (%)	Age in years, mean (SD)	Cancer type(s)
Bantum, 2014 [61]	USA	RCT	303	86.1% (303/352)	82	49.3 (11)	Any type of cancer, completed treatment >4 weeks prior to study
Berg, 2014 [62]	USA	Pre-post	19	79.2% (19/24)	71	23.4 (3.9)	Adult (18–34 years) survivors of childhood cancers (any type)
Forbes, 2015 [63]	Canada	RCT	87	91.6% (87/95)	56	65.1 (8.5)	Breast, prostate and colorectal cancer survivors (96% currently disease free; 75% completed treatment)
Hatchett, 2013 [55]	USA	RCT	74	87.1% (74/95)	100	No data	Breast cancer survivors, completed treatment
Hoffman, 2014 [64]	USA	Pre-post	7	100% (7/7)	71	64.6 (6.5)	NSCLC survivors (immediately before + after surgery/during treatment)
Hong, 2015 [65]	USA	Pre-post	26	86.7% (26/30)	69	69 (median)	Any type of cancer survivor, either undergoing or completed treatment
Kanera, 2016 [71]	Netherlands	RCT	394^a^	89.2% (462/518)^a^	80	56.0 (11.4)	Any type of cancer, completed treatment >4 and <56 weeks prior to study, no recurrence
Kuijpers, 2016 [66]	Netherlands	Pre-post	73	79.3% (73/92)	100	49.5 (11.4)	Breast cancer survivors, either undergoing or completed treatment 3–12 months prior to study
Lee, 2014 [67]	South Korea	RCT	57	96.6% (57/59)	100	43.2 (5.1)	Breast cancer survivors, completed treatment <1 year prior to study
McCarroll, 2015 [68]	USA	Pre-post	35	70.0% (35/50)	100	58.4 (10.3)	Overweight/obese breast and/or endometrial cancer survivors with desire to lose weight, diagnosis <3 years prior to study
Puszkiewicz, 2016 [69]	UK	Pre-post	11	100% (11/11)	82	45 (9.4)	Breast, prostate or colorectal cancer survivors, completed treatment
Quintiliani, 2016 [70]	USA	Pre-post	10	100% (10/10)	100	58.6 (6.1)	Breast cancer survivors, >2 years since diagnosis and >6 months since end of treatment
Rabin, 2012 [54]	USA	RCT	17	94.4% (17/18)	56	32.2 (5.6)	Young adult (18–39) cancer survivors, completed treatment <10 years prior to study
Short, 2016 [72]	Australia	Pre-post^b^	156^b^	31.7%^c^ (156/492)	100	55.0 (9.7)	Breast cancer survivors, completed treatment
Valle, 2013 [53]	USA	RCT	66	76.7% (36/86)	91	31.7 (5.1)	Young adult (21–39) cancer survivors, diagnosed >18 years of age, >1 year since diagnosis, completed treatment

^a^Kanera et al. presented physical activity data for 394 participants (sample size used in meta-analysis); however, retention for other measures at follow-up was 462

^b^Short et al. (2016) is treated as a pre-post study due to the lack of control group across the three intervention arms

^c^Short et al.’s paper presents results for 3-month follow-up (immediately post-intervention) because retention rate at 6 months was very low (10.8% (53/492)). Sample size/retention rate presented here is for the 3-month follow-up

**Table 2:** Intervention types and outcomes for included studies

Author, year	Intervention type	Study duration	Description of intervention	Behaviour change techniques (BCTs)	Approaches to measurement of engagement/adherence	Control group treatment	Outcomes measured
Bantum, 2014 [61]	Online workshop (website)	6 months	6-week online course providing information, skill building, weekly action plans, social networking and peer support, exercise logs, relaxation exercises	1.1 Goal setting (behaviour) 1.2 Problem solving 1.4 Action planning 2.3 Self-monitoring of behaviour 3.1 Social support (unspecified) 9.1 Credible source	Mean (SD) online sessions attended was 5.3 (1.28) 67% attended all 6 sessions 86.8% attended >4 sessions	Waitlist control	PA, diet, fatigue, depression, insomnia
Berg, 2014 [62]	Emails + associated website	12 weeks	Biweekly emails to deliver module content and website provides graphical depictions of participant-reported health behaviours and health information.	2.2 Feedback on behaviour 2.3 Self-monitoring of behaviour Participants also offered deals for healthy goods/services in local area for completing self-monitoring (regardless of behaviour reported)	Completion of self-monitoring (‘check-in assessment’) over the 12-module period (6 weeks) fell from 91.7 to 66.7%	N/A	PA, SE, alcohol consumption, smoking
Forbes, 2015 [63]	Online workshop (website)	10 weeks	9-week workshop to deliver content (e.g. dispelling PA myths, exercising safely, planning/making SMART goals). Website used to log/monitor PA and email feedback.	2.2 Feedback on behaviour 2.3 Self-monitoring of behaviour 5.1 Information about health consequences 6.2 Social comparison 9.1 Credible source 10.4 Social reward	Percentage of completed modules fell from 50% (week 1) to 10% (week 9) 94% logged in at least once, 85% recorded PA at least once, 67% viewed modules at least once	Waitlist control	PA, QoL (cancer-specific), QoL (generic), fatigue
Hatchett, 2013 [55]	Email + access to e-counsellor	12 weeks	Emails designed to increase PA by influencing SCT variables. E-counsellor offered tailored PA advice and encouraged participant engagement with intervention.	1.1 Goal setting (behaviour) 1.2 Problem solving 2.3 Self-monitoring of behaviour 3.1 Social support (unspecified) 9.1 Credible source 13.5 Identity associated with changed behaviour	Not measured/reported	Waitlist control	PA, SE, self-regulation, OE value, exercise role identity
Hoffman, 2014 [64]	Nintendo Wii Fit Plus	16 weeks	Virtual walking environment with gradual increase in target walking time. Three Wii Fit Plus balance exercises per day were also recommended.	1.1 Goal setting (behaviour) 1.5 Review behaviour goals 2.3 Self-monitoring of behaviour 8.7 Graded tasks 9.1 Credible source 12.5 Adding objects to the environment	Mean (SD) adherence rate to intervention at end of study was 87.6% (12.2%)	N/A	PA, fatigue, SE
Hong, 2015 [65]	Mobile-enabled website	8–12 weeks	Website used for goal setting, activity tracking, personalised feedback and progress reviews, social networking, tips on healthy living and links to reliable health information	1.1 Goal setting (behaviour) 1.4 Action planning 1.5 Review behaviour goals 1.6 Discrepancy between current behaviour and goal 2.2 Feedback on behaviour 2.3 Self-monitoring of behaviour 3.1 Social support (unspecified) 5.1 Information about health consequences 9.1 Credible source	Website use: 12% once/fortnight; 62% once/week; 19% 2–3 times/week 8% 4–5 times/week	N/A	PA, QoL (generic)
Kanera, 2016 [71]	Online workshop (website)	6 months	Automated system to evaluate baseline assessment and select personalised intervention components using ‘if-then’ algorithms. Users are recommended modules based on assessments but have access to all 8 modules.	1.1 Goal setting (behaviour) 1.2 Problem solving 1.4 Action planning 1.5 Review behaviour goals 1.6 Discrepancy between current behaviour and goal 2.2 Feedback on behaviour 2.3 Self-monitoring of behaviour 3.1 Social support (unspecified) 5.1 Information about health consequences 6.2 Social comparison 7.1 Prompts/cues 8.2 Behaviour substitution 9.1 Credible source 9.2 Pros and cons 11.2 Reduce negative emotions 13.2 Framing/reframing	Specific modules were recommended to participants based on current behaviours from 8 modules in total. Participants followed mean (SD) 2.23 (1.58) modules. 25% participants followed PA module, and 62% followed diet module.	Waitlist control	PA, diet, smoking
Kuijpers, 2016 [66]	Online portal (website)	4 months	Offers personalised education materials, overview of appointments, access to EMR. Tailored PA support based on clinical characteristics, PA levels and motivation.	1.1 Goal setting (behaviour) 2.2 Feedback on behaviour 2.3 Self-monitoring of behaviour 6.2 Social comparison 9.1 Credible source	Website logins ranged from 0 to 62, and duration of use ranged from 2 to 38 min. Participants on treatment (M = 10.9 logins) used the website more often than off-treatment (M = 5.6 logins) participants but those who were off treatment had a longer mean duration (15.2 min) of log in compared to those on treatment (11.3 min)	N/A	PA, QoL (generic), SE
Lee, 2014 [67]	Website + text messaging	12 weeks	Website used for assessment, education, tailored information provision and action planning (goal setting, scheduling, monitoring and automatic feedback). Daily automatic feedback provided on recommended and actual behaviours	1.1 Goal setting (behaviour) 1.4 Action planning 1.6 Discrepancy between current behaviour and goal 2.2 Feedback on behaviour 2.3 Self-monitoring of behaviour 5.1 Information about health consequences 5.6 Information about emotional consequences 7.1 Prompts/cues 9.1 Credible source	Not measured/reported	50-page booklet on exercise/diet	PA, diet, QoL (cancer-specific), fatigue, anxiety, depression, SE
McCarroll, 2015 [68]	Mobile app	4 weeks	Participants log nutrition/PA through app which provided real-time personalised feedback. Limited carbohydrate intake to <70 g/day and increase fibre intake to 30 g/day.	1.1 Goal setting (behaviour) 1.3 Goal setting (outcome) 2.2 Feedback on behaviour 2.3 Self-monitoring of behaviour 2.4 Self-monitoring of outcomes of behaviour 3.1 Social support (unspecified) 7.1 Prompts/cues 9.1 Credible source 15.1 Verbal persuasion about capability	Not measured/reported	N/A	PA, diet, QoL (cancer-specific), SE, weight, waist circumference, BMI
Puszkiewicz2016 [69]	Mobile app	6 weeks	Tailored PA programme using video demonstrations is recommended based on users’ preferred PA goals, duration, type and difficulty of PA.	1.1 Goal setting 1.4 Action planning 2.2 Feedback on behaviour 2.3 Self-monitoring of behaviour 4.1 Instruction on how to perform a behaviour 6.1 Demonstration of the behaviour 7.1 Prompts/cues 8.7 Graded tasks 10.4 Social reward	Participants used app mean (SD) of 2.07 (0.68) times per week. Mean (SD) session duration was 25.08 (8.22) minutes. Mean (SD) app use duration was 44.00 min (20.50) per week (range 24.50–91.00 min).	N/A	PA, QoL (cancer-specific and generic), fatigue, BMI, anxiety, depression, sleep quality
Quintiliani, 2016 [70]	Text messaging + Fitbit + telephone counselling	10 weeks	Text messages assess participants’ dietary intake, and Fitbit assesses weight and step count. Four technology-assisted telephone calls (based on PA, sleep and 2 nutrition-related topics) were guided by motivational interviewing	1.1 Goal setting (behaviour) 1.5 Review behaviour goals 1.6 Discrepancy between current behaviour and goal 2.2 Feedback on behaviour 2.3 Self-monitoring of behaviour 2.4 Self-monitoring of outcome of behaviour 7.1 Prompts/cues 9.1 Credible source	Of 70 opportunities (7 days/week × 10 weeks), mean (SD) responses to text messages was 60 (13), recording a step measurement was 64 (7), recording a weight measurement was 45 (24) and recording a sleep measurement was 43 (19). All participants completed all 4 counselling calls.	N/A	PA, diet, fatigue, perceived stress, SE, weight
Rabin, 2012 [54]	Website	12 weeks	PA manual provided matched with participants’ ‘stage of change’ and responses to questionnaires on the website. Website also enabled participant to set PA goals and log PA	1.1 Goal setting (behaviour) 2.2 Feedback on behaviour 2.3 Self-monitoring of behaviour 3.1 Social support (unspecified) 5.1 Information about health consequences 9.1 Credible source	Mean (SD) website login was 14.75 (8.46). Mean (SD) number of days participants logged PA was 11.38 (7.93), used goal setting feature was 5.25 (4.17), used stage-based manual was 3.13 (2.17), accessed PA-related information was 1.25 (1.28), accessed PA resources was 0.88 (1.13) and accessed PA tips was 0.50 (1.07).	Provided with 3 cancer + survivorship websites	PA, fatigue, POMS,
Short, 2016 [72]	Online workshop (website)	12 weeks	3 online modules delivered with a combination of non-tailored information (PA guidelines, increasing motivation and preventing relapse) and tailored support (e.g. based on current PA, outcome expectations, health status, eliciting social support, overcoming barriers, action planning)	1.1 Goal setting (behaviour) 1.2 Problem solving 1.4 Action planning 1.5 Review behaviour goals 1.6 Discrepancy between current behavioural and goal 2.2 Feedback on behaviour 2.3 Self-monitoring of behaviour 3.1 Social support (unspecified) 4.1 Instruction on how to perform a behaviour 5.1 Information about health consequences 5.2 Salience of consequences 7.1 Prompts/cues 8.7 Graded tasks 9.1 Credible source	Mean (SD) website usage duration was 61.1 min (80.1) (range 0–550 min). Mean (SD) website login was 5.18 (8.48) (range 1–45). All participants viewed at least one module. 85% in the weekly module group and 73% in the monthly module group viewed all 3 modules. 60% of the weekly module group completed 2 (of 3) modules compared to 46% of the monthly module group. 75% of participants completed at least one action plan. 91.7% in the monthly module group completed 2 action plans compared to 71.7% in weekly module group.	N/A	PA
Valle, 2013 [53]	Facebook support group + website	12 weeks	Weekly messages (sent via Facebook) and group administrator posted discussions within the group throughout intervention. Participants also had access to website for goal setting and PA diary and pedometers to measure steps.	1.1 Goal setting (behaviour) 1.2 Problem solving 1.6 Discrepancy between current behavioural and goal 2.2 Feedback on behaviour 2.3 Self-monitoring of behaviour 3.1 Social support (unspecified) 5.1 Information about health consequences 9.1 Credible source 12.5 Adding objects to the environment	Intervention participants posted a total of 153 Facebook comments to group wall compared to 188 comments in control group. 49% of participants in both groups made >2 Facebook posts in the study period. Intervention participants set a mean of 4.2 goals, and submitted a mean of 21.9 PA entries and 13.1 steps entries. 71% tracked PA data at least once. Proportion of participants logging either PA or steps declined from 57.8% in week 1 to 24.4% in week 12.	Basic Facebook group membership	PA, QoL (cancer-specific), BMI

^a^Short et al. (2016) is treated as a pre-post study due to the lack of a control group across the three intervention arms (single module, three weekly modules, three monthly modules)

*PA* physical activity, *SE* self-efficacy, *SMART* specific, measurable, attainable, realistic, timely, *SCT* social cognitive theory, *OE* outcome expectancy, *QoL* quality of life, *EMR* electronic medical record

## Primary outcomes

### Physical activity and sedentary time

All 15 included studies measured the impact of DBCIs on PA [53–55, 61–72]. All used self-reported PA as outcomes: five used the Godin Leisure-Time Exercise Questionnaire (GLTEQ) [53, 61, 63, 69, 72], two the International Physical Activity Questionnaire (IPAQ) [66, 70], one a 7-day PA recall [54] and one the Short Questionnaire to Assess Health Enhancing Physical Activity (SQUASH) [71]; two identified the number of days in the last seven that the participant engaged in moderate and/or vigorous PA [55, 62]; three studies reported percentage of participants meeting PA guidelines (150 min of MVPA per week) [54, 63, 67] and two reported stages of change for PA [65, 67]. Short et al. [72] also reported a resistance training score. Hoffman et al. [64] reported the number of minutes walked, steps walked and number of balance exercises completed. McCarroll et al. [68] reported number of minutes of PA completed and the number of calories expended as logged via the participant using the mobile app used for their intervention.

**Fig. 2:**
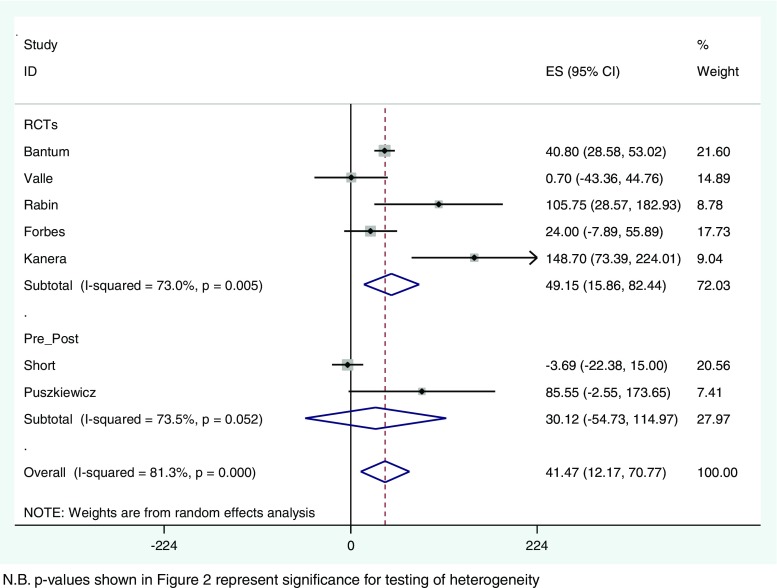
Meta-analysis of DBCIs on MVPA

MVPA (minutes) was available for 11 studies (five as a combined variable [53–55, 61, 72], five as separate moderate and vigorous variables (combined for the purposes of the meta-analysis) [62, 63, 66, 70, 71], and raw data was available for Puszkiewicz et al. [69] to calculate a combined MVPA variable). Of these, seven (five RCTs [53, 54, 61, 63, 71] and two pre-post studies [69, 72]) reported MVPA duration in minutes per week and were pooled in a random effects meta-analysis using data from 1034 participants (see Fig. 2). DBCIs resulted in significant increases in MVPA minutes/week (MD = 41; 95% CI 12, 71; *p* = 0.006) with very high levels of heterogeneity (*I* ^2^ = 81%). Independently, the RCTs showed a significant increase in MVPA (MD = 49, 95% CI 16, 82, *p* = 0.004, *I* ^2^ = 73%). A funnel plot suggested that there may be some indication of publication bias among smaller studies (see Fig. 1, Online Resource 2).

Of the other eight studies which could not be included in the meta-analysis, four reported a significant effect, [55, 65–67], two did not report significant findings [62, 68] and two did not conduct significance testing due to small sample sizes [64, 70]. No studies reported effects on sedentary time.

### Diet

Five studies measured the impact of DBCIs on dietary intake [61, 67, 68, 70, 71]. Due to the variation in approaches to assessment and measurement of dietary outcomes, a meta-analysis was not considered appropriate. Three studies [61, 67, 71] were RCTs and two were pre-post studies [68, 70]. Only two of the studies reported a significant effect on dietary outcomes [67, 71]; however, this no longer remained significant after correcting for multiple testing in Kanera et al.’s study [71]. Quintiliani et al. [70] did not conduct significance testing, due to the very small sample (*n* = 10).

## Secondary outcomes

### BMI/weight

Four studies assessed BMI and/or weight (one RCT [53] and three pre-post studies [68–70]). Three assessed BMI [53, 68, 69] and Quintiliani et al. assessed weight [70]. Using data from 122 participants (66 participants in RCTs; 56 in pre-post studies), there was a significant pooled reduction in BMI/weight (SMD = −0.23; 95% CI −0.41, −0.05; *p* = 0.011; *I* ^2^ = 0.0%) (see Fig. 2, Online Resource 2). The RCT showed a significant reduction in BMI (SMD = −0.28, 95% CI −0.52, −0.04, *p* = 0.023). A funnel plot revealed no evidence of publication bias for BMI/weight outcomes.

### Other cancer-relevant outcomes

#### Fatigue

**Fig. 3:**
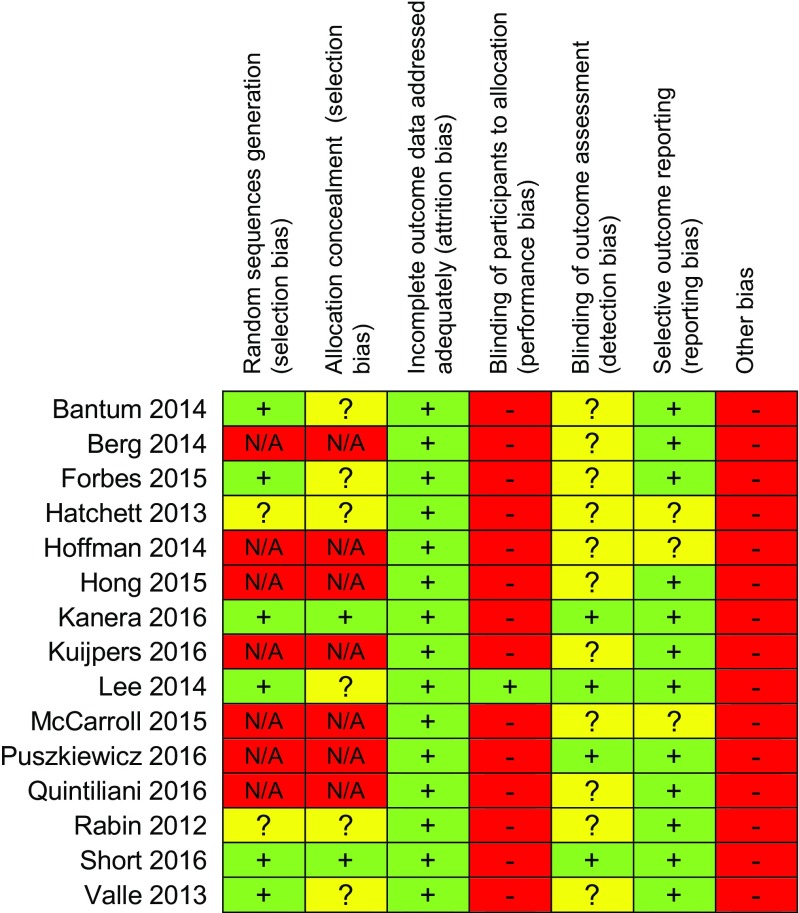
Risk of bias in included studies

Seven studies measured the impact of DBCIs on fatigue [54, 61, 63, 64, 67, 69, 70]. Of these, three used the Brief Fatigue Inventory (BFI) [61, 64, 67], two used the Functional Assessment of Chronic Illness Therapy-Fatigue (FACIT-F) [63, 69], one used the Profile of Mood States-Fatigue (POMS-Fatigue) [54] scale and one used a 0–10 scale [70]. SMDs were required to pool effects across studies due to the variation in measurement tools. Therefore, meta-analysis was only conducted on the three RCTs where appropriate data could be extracted [54, 61, 63], using data from 406 participants (see Fig. 3, Online Resource 2). DBCIs resulted in a decrease in fatigue, but this was not significant (SMD = −0.23; 95% CI −0.51, 0.05; *p* = 0.103; *I* ^2^ = 78%). Once again, very high levels of heterogeneity were displayed for fatigue. A funnel plot revealed no evidence of publication bias for fatigue outcomes. Of the remaining four studies, only one reported a significant reduction in fatigue [67]. Two of these studies did not report significance testing [64, 70] due to very small sample sizes (7 and 10, respectively).

#### Cancer-specific QoL

Five studies assessed cancer-specific measures of QoL [53, 63, 67–69]. Four studies used the FACT-G [53, 63, 68, 69] and one used the Quality of Life Questionnaire-Core 30 (QLQ-C30) [67]. The four studies using the FACT-G (two RCTs [53, 63] and two pre-post studies [68, 69]) were pooled using data from 198 participants (152 participants in RCTs; 46 from pre-post studies) (see Fig. 4, Online Resource 2). Overall, there were no significant changes on cancer-specific QoL (MD = 0.61; 95% CI −1.83, 3.06; *p* = 0.62; *I* ^2^ = 42%). Similar results are shown when just pooling results from RCTs (MD = 0.06; 95% CI −2.44, 2.57; *p* = 0.960; *I* ^2^ = 0%). A funnel plot revealed no evidence of publication bias for cancer-specific QoL outcomes. The remaining study also found a non-significant difference between groups [67].

#### Generic QoL

Four studies assessed generic measures of QoL [63, 65, 66, 69]. Each study reported various domains of QoL as opposed to a global score using various measurement tools (i.e. Short Form (36) Health Survey (SF-36) [63, 66], the EuroQol 5 Dimensions (EQ5D) [69] and a seven-item non-validated measure [65]). Two studies found no significant changes in any QoL domains [63, 69]. One study found a significant improvement in role functioning-emotional and mental health for those during treatment and a significant improvement in social functioning for those who had finished treatment [66]. Hong et al. found significant improvements in self-rated health, fatigue, pain, shortness of breath, stress, sleep quality and overall QoL using a non-validated scale [65].

#### Mental health

Three studies measured the impact of DBCIs on depression [61, 67, 69], none of which reported any significant impact. Two studies assessed the impact on anxiety [67, 69], neither of which reported a significant effect. Rabin et al. [54] also measured Profile of Mood States (anger, depression, tension/anxiety, vigour, confusion) and did not find a significant change in scores between groups.

#### Sleep disturbance

Two studies measured the impact of DBCIs on sleep disturbance [61, 69]. Both studies reported a significant improvement in sleep quality; Bantum et al. [61] showed a significant reduction in insomnia, and Puszkiewicz et al. [69] showed a significant improvement in sleep quality.

### Theoretical underpinning

Twelve studies reported some level of theoretical basis to their intervention design [53–55, 62, 64–68, 70–72]. Of those that did mention a theoretical influence, Social Cognitive Theory (SCT) was most frequently reported [53, 55, 66, 68, 71, 72], and sometimes used in combination with other theories (i.e. Transtheoretical Model (TTM) [54] or the Theory of Planned Behaviour (TPB) [66]). Other theories included the Theory of Reasoned Action [62], Theory of Symptom Self-Management [64], Theory of Goal Setting [65], the TTM alone [67] and the Social Contextual Model [70]. The description of the theoretical underpinning of the DBCIs varied across studies, but was relatively poor. Only seven studies explicitly reported how theory/predictors were used to select/develop intervention techniques [53–55, 66, 67, 71, 72], and only six studies used theory/predictors to tailor intervention techniques to participants [54, 64, 66, 67, 71, 72]. Six studies measured theory-relevant constructs and reported outcomes pre and post intervention [53, 62, 66–68, 70]. Of these six studies, three reported no significant change in measures related to self-efficacy [62, 66, 70]. Lee et al. [67] reported that the ‘stage of change’ and self-efficacy for PA and fruit and vegetable consumption was significantly higher in the intervention group, and McCarroll et al. [68] reported a significant increase in self-efficacy between pre and post intervention. Valle et al. was the only study which conducted mediational analysis of theoretical constructs/predictors (presented in an associated paper [73]). However, this study showed that the intervention group reported lower self-efficacy for PA and social support from friends on social networking websites compared to the control group. Changes in social support from friends on social networking sites were positively related to changes in MVPA; however, it was those in the control group who reported increased social support from friends on social networking sites compared to the intervention group [73].

### Risk of bias in included studies

See Fig. 3 for an assessment of the risk of bias in the included studies. Study quality was deemed to be low for the majority of included studies. For the RCTs, there was adequate randomisation sequence generation in the majority of the studies; however, allocation concealment was much less clear. The lack of a control group in the seven pre-post studies highly increased the risk of bias, reducing the confidence which can be placed on the observed effects. All of the studies were judged to be high risk for other sources of bias, for instance all 15 studies used self-reported PA, as opposed to objective measures (e.g. accelerometry). Some of the RCTs did not report (or it was unclear) whether baseline outcome measures and/or participant characteristics were similar between groups [53, 54, 61, 63, 71, 72]. Furthermore, the representativeness of the sample was fairly narrow in that the majority of the study samples which consisted of predominantly female, middle-aged, English-speaking, breast cancer survivors of high educational/income level, which likely limits the generalizability of the findings across the wider population of cancer survivors. Most of the studies had small sample sizes (generally ranging between 20 and 100), and only three studies had larger samples [61, 71, 72] (303, 462 and 492, respectively). Despite the largest sample size at baseline in Short et al.’s study [72], retention was extremely low (32% at 3 months and 11% at 6 months of follow-up).

## Discussion

The current meta-analysis found that DBCIs resulted in an increase in MVPA participation of approximately 40 min per week. While meta-analysis was not possible for dietary outcomes, there was mixed evidence for an effect on dietary intake. No studies assessed sedentary behaviour. Meta-analyses also revealed a significant reduction in BMI, a reduction in fatigue which did not reach statistical significance, and no change was seen in cancer-specific measures of QoL. For other secondary outcomes where meta-analysis was not possible, there was mixed evidence for the effect on domains of generic QoL measures and theoretical constructs (e.g. self-efficacy). There is no evidence for an improvement in anxiety or depression, and while only two studies assessed sleep disturbance, both reported a significant improvement.

To our knowledge, this is the first meta-analysis to assess the current evidence with regards to DBCIs targeting PA and/or diet among cancer survivors. An increase in approximately 40 min of MVPA per week is important given that this represents a substantial proportion (27%) of cancer survivors’ recommended weekly MVPA participation [23–25]. While there is limited evidence on the dose-response relationship between MVPA and mortality and recurrence outcomes, Schmid et al. estimated that each 10 MET-hour per week increase in post-diagnosis PA (approximately equivalent to the 150-min MVPA/week guideline) was associated with 24% (95% CI 11–36%) and 28% (95% CI 20–35%) decreased total mortality risk for breast and colorectal cancer survivors, respectively [18]. Schmid et al. also reported that breast or colorectal cancer survivors who increased their PA by any amount between pre and post diagnosis showed a decreased total mortality risk (RR = 0.61, 95% CI 0.46–0.80) compared to cancer survivors who did not change their PA level or who were insufficiently active pre diagnosis [18]. Therefore, even small increases in MVPA post diagnosis are likely to be beneficial for cancer survivors.

It is noteworthy that a third of the included studies were published in 2016, illustrating the rise in research interest of the effectiveness of DBCIs. It is interesting to compare the findings of this study with other meta-analyses using non-digital PA interventions among cancer survivors. While we did not find a significant reduction in fatigue (SMD = −0.23), Mishra et al. did find a significant improvement, both for survivors who had completed treatment (SMD = −0.82) [19] and who were still undergoing active treatment (SMD = −0.73) [74]. We also did not find any improvement in cancer-specific measures of QoL, while Mishra et al. reported positive improvements in both of their meta-analyses. Mishra et al. also found significantly improved anxiety and depression, sleep quality and improvements in some domains of generic measures of QoL (e.g. social, physical and role functioning) [19, 74]. The small number of studies assessing these outcomes meant that meta-analyses for these outcomes were not possible in the current study. However, in the studies that did assess these outcomes, we did not find any evidence for an improvement in anxiety and depression, but both studies assessing sleep reported significant improvements. We also found mixed evidence for individual domains of generic QoL measures. It is possible that the non-digital interventions included in Mishra et al.’s studies result in larger effect sizes as many of the interventions are supervised by trained staff or involve some level of human interaction, which may foster higher levels of engagement and adherence to the intervention. However, due to the small number of low-quality studies included in this review, there is a need for more high-quality RCTs, with objective measures of PA, long-term follow-up and larger sample sizes before reliable comparisons between non-digital and DBCIs can be made.

Sustained engagement with DBCIs was a significant problem for a number of the studies included in this review. For instance, in the study conducted by Short et al., retention at the 3-month follow-up was only 32% (156/492) and 11% (53/492) at the 6-month follow-up [72]. Furthermore, while 75% of the sample completed at least one action plan, the average number of action plans completed was only 2.2. Similarly, 50% of participants completed the week 1 module compared to 10% for the week 9 module in Forbes et al.’s study [63]. A systematic review has shown that there is a positive relationship between participants’ adherence to/engagement with digital interventions and positive physical health outcomes across a range of populations and behaviours, suggesting that efforts to improve effective engagement with DBCIs could improve behaviour change outcomes [75]. It is possible that suboptimal engagement with the DBCIs in the studies included in this review may explain the reduced effects on outcomes compared to those observed in Mishra et al.’s review of non-digital PA interventions [19, 74]. Future DBCI studies should integrate techniques or components that maintain effective engagement with the intervention for its duration. There is some evidence that technology-based strategies (e.g. reminders, prompts) can encourage user engagement [76]. Other aspects which have been identified as important for engagement include ease of use, design aesthetic, feedback, function, ability to change design to suit own preferences, tailored information and unique mobile phone features [77]. Similarly, less time consumption, user-friendly design, real-time feedback, individualised elements, detailed information and health professional involvement may also improve effectiveness of DBCIs, in particular mobile apps [78]. Furthermore, future studies should aim to better understand the link between engagement and effectiveness of DBCIs targeting PA and diet in cancer survivors and define, evaluate and report engagement more consistently so as to better understand techniques that foster effective engagement and mechanisms of action [79].

The majority (10/15) of the studies used an online portal or website to deliver the intervention, and, while one of these websites was mobile-enabled [65], only two studies used mobile apps [68, 69]. This is interesting given the findings of a recent review of 23 interventions using mobile apps that found that 17 of the included studies reported a significant effect on behaviour change in the general population [78]. It is possible that interventions using mobile apps may be more effective than other types of DBCIs due to the widespread usage of and constant access to smartphones and the Internet. The most recent Ofcom Communications Market report conducted in the UK reports that 71% of UK adults own a smartphone and 66% use their smartphone to access the Internet most frequently [41]. Therefore, it would be interesting for future studies to use mobile apps as a mode of intervention delivery and compare the effectiveness of mobile app interventions compared to other DBCIs as they may foster higher levels of engagement.

Self-monitoring, goal-setting, credible source and feedback on behaviour were the most frequently described BCTs used in the included studies. Due to the heterogeneity in intervention type, mode of delivery, behavioural outcomes and measurement approaches, it is difficult to interpret which BCTs were most effective at changing PA/dietary behaviour. Kanera et al.’s study used the most BCTs (*n* = 16) but also used a tailored if-then algorithm within the intervention to automatically tailor content to participants; therefore, it is difficult to ascertain what intervention components or approaches to delivery lead to increased effectiveness. A recent meta-analysis revealed that theory-based interventions are significantly more effective at improving PA [80]. SCT was the most commonly reported theoretical basis of the interventions; however, several other behaviour change theories were used across the studies. The level of reporting of the extent to which theory was incorporated into the development of the interventions varied across studies, but was generally poor. Only three studies did not report any theoretical underpinning. Future studies should aim to explicitly report how theory is used to develop intervention techniques and tailor the intervention to participants. Measurement and exploration of changes in targeted theoretical constructs (e.g. via mediation analyses) can aid understanding of why interventions may or may not be effective [59] and can be used to refine theoretical models of behaviour change. The lack of clarity about the intervention content and theoretical underpinning and the lack of measurement on theoretical constructs mean it is difficult to unpick how or why the interventions which improved behaviour in this review were effective. Future DBCI studies should clearly report any theoretical underpinning and behaviour change techniques used, for instance by using Michie et al.’s Theory Coding Scheme [59] and Behaviour Change Technique Taxonomy [56].

There are several limitations to this review. Primarily, the data extracted for the meta-analyses reflect unadjusted models. While the forest plot for MVPA minutes/week (Fig. 2) illustrates that Bantum et al. [61], Rabin et al. [54] and Kanera et al. [71] found a significant effect, the original publications show that this no longer remained significant when adjusting for baseline values and/or other covariates (e.g. demographics, disease characteristics) [54, 61], or when adjusted models are controlled for multiple testing [71]. Bantum et al. [61] did find a significant increase in vigorous PA participation in the adjusted model, but not when combined with moderate PA. Similar issues arise for the meta-analyses for fatigue and BMI/weight. The studies that show significant findings for fatigue and BMI/weight in the current meta-analyses do not report significant findings when adjusted for baseline and/or covariates in the original publications. Therefore, pooling the adjusted results would reduce the overall effect size substantially, and it is likely that this would no longer remain significant. Furthermore, a combined MVPA variable was chosen to assess PA outcomes to maximise the number of studies that could be included in the meta-analysis and to coincide with the American College of Sports Medicine’s recommendation that cancer survivors should follow the PA guidelines for the general population (at least 150 min of at least moderate intensity PA per week) [25]. However, this means the current meta-analysis does not differentiate between different intensities of PA: it may be beneficial to evaluate the effect on outcomes at different intensities of PA. Visual inspection of funnel plots suggested possible publication bias for smaller studies assessing MVPA outcomes; it is possible that our choice to only include published studies may have increased publication bias. There was no suggestion of publication bias for BMI/weight, fatigue or cancer-specific QoL measures.

The risk of bias in included studies was high. The inclusion of one-arm, pre-post studies substantially increases the risk of bias; however, we felt that the novelty of this area of research warranted the inclusion of these studies and that this added valuable insight into the current state of the literature. Few studies assessed outcomes other than PA, where only half of the studies could be included in a meta-analysis. Heterogeneity across studies was very high, likely due to the variability of types of DBCIs, intervention content, cancer type and populations, outcome measurement tools, etc. With the addition of future studies, more specific inclusion criteria could be used to assess effectiveness of more similar studies. All of the PA findings used self-report data, which while easy to use, inexpensive and validated, often hugely underestimate or overestimate PA participation [81]. Therefore, we advise that these results are interpreted with caution until the number of published studies assessing DBCIs increases and inclusion criteria for future systematic reviews can be more stringent for low-quality trials.

To the best of our knowledge, this is the first meta-analysis of DBCIs designed to improve PA and diet among cancer survivors. While the review shows some evidence of an improvement in MVPA, a reduction in BMI and a trend towards significance for fatigue, large, high-quality RCTs, with objective measures of PA and long-term follow-up, are lacking. Future studies should aim to address these limitations, but the approach of using digital technology in this context appears promising.

## Electronic supplementary material

Online Resource 1 (DOCX 51 kb)

Online Resource 2 (DOCX 51 kb)
