# Supplementary material for: Digital health behaviour change interventions targeting physical activity and diet in cancer survivors: a systematic review and meta-analysis
Source: J Cancer Surviv. 2017 Aug 4;11(6):704–19. doi: 10.1007/s11764-017-0632-1 (PMC5671545; doi:10.1007/s11764-017-0632-1)
Supplement: Supplementary file 1 — (DOCX 51 kb) [file 11764_2017_632_MOESM1_ESM.docx]

Article title: Digital health behaviour change interventions targeting physical activity and diet in cancer survivors: a systematic review and meta-analysis

Journal: Journal of Cancer Survivorship

Author names: Anna L Roberts, Abigail Fisher, Lee Smith, Malgorzata Heinrich, Henry W W Potts

Corresponding author: Abigail Fisher, Department of Behavioural Science & Health, University College London, Gower Street, London, WC1E 6BT, United Kingdom.

Email: [abigail.fisher@ucl.ac.uk](mailto:abigail.fisher@ucl.ac.uk)

Search Strategy/Search Terms:

Medline (Ovid)

| 1. physical activity.mp. or exp Motor Activity/ |  |
| --- | --- |

| 2. limit 1 to (english language and humans) |  |
| --- | --- |

| 3. physical fitness.mp. or exp Physical Fitness/ |  |
| --- | --- |

| 4. limit 3 to (english language and humans) |  |
| --- | --- |

| 5. fitness.mp. |  |
| --- | --- |

| 6. limit 5 to (english language and humans) |  |
| --- | --- |

| 7. exp Exercise/ or exp Exercise Therapy/ or exercise*.mp. |  |
| --- | --- |

| 8. limit 7 to (english language and humans) |  |
| --- | --- |

| 9. exercise therap*.mp. |  |
| --- | --- |

| 10. limit 9 to (english language and humans) |  |
| --- | --- |

| 11. diet*.mp. or exp Diet/ or exp Diet Therapy/ |  |
| --- | --- |

| 12. limit 11 to (english language and humans) |  |
| --- | --- |

| 13. exp Food Habits/ or exp Energy Intake/ or dietary quality.mp. |  |
| --- | --- |

| 14. limit 13 to (english language and humans) |  |
| --- | --- |

| 15. sedentary lifestyle*.mp. or exp Life Style/ |  |
| --- | --- |

| 16. limit 15 to (english language and humans) |  |
| --- | --- |

| 17. exp Sedentary Lifestyle/ or exp Health Behavior/ or sedentary behavio?r*.mp. |  |
| --- | --- |

| 18. limit 17 to (english language and humans) |  |
| --- | --- |

| 19. sedentary time.mp. |  |
| --- | --- |

| 20. limit 19 to (english language and humans) |  |
| --- | --- |

| 21. lifestyle.mp. |  |
| --- | --- |

| 22. limit 21 to (english language and humans) |  |
| --- | --- |

| 23. health behavio?r*.mp. |  |
| --- | --- |

| 24. limit 23 to (english language and humans) |  |
| --- | --- |

| 25. exp Health Promotion/ or behavio?r change*.mp. |  |
| --- | --- |

| 26. limit 25 to (english language and humans) |  |
| --- | --- |

| 27. 2 or 4 or 6 or 8 or 10 or 12 or 14 or 16 or 18 or 20 or 22 or 24 or 26 |  |
| --- | --- |

| 28. self management.mp. or exp Self Care/ |  |
| --- | --- |

| 29. limit 28 to (english language and humans) |  |
| --- | --- |

| 30. intervention.mp. |  |
| --- | --- |

| 31. limit 30 to (english language and humans) |  |
| --- | --- |

| 32. sitting.mp. |  |
| --- | --- |

| 33. limit 32 to (english language and humans) |  |
| --- | --- |

| 34. exp Body Weight/ or exp Diet, Reducing/ or exp Weight Loss/ or weight control.mp. |  |
| --- | --- |

| 35. limit 34 to (english language and humans) |  |
| --- | --- |

| 36. body weight.mp. |  |
| --- | --- |

| 37. limit 36 to (english language and humans) |  |
| --- | --- |

| 38. weight reduction.mp. |  |
| --- | --- |

| 39. limit 38 to (english language and humans) |  |
| --- | --- |

| 40. weight loss.mp. |  |
| --- | --- |

| 41. limit 40 to (english language and humans) |  |
| --- | --- |

| 42. exp Health Education/ |  |
| --- | --- |

| 43. limit 42 to (english language and humans) |  |
| --- | --- |

| 44. 29 or 31 or 33 or 35 or 37 or 39 or 41 or 43 |  |
| --- | --- |

| 45. exp Health Knowledge, Attitudes, Practice/ |  |
| --- | --- |

| 46. limit 45 to (english language and humans) |  |
| --- | --- |

| 47. 44 or 46 |  |
| --- | --- |

| 48. 45 or 46 or 47 |  |
| --- | --- |

| 49. exp Eating/ |  |
| --- | --- |

| 50. limit 49 to (english language and humans) |  |
| --- | --- |

| 51. 2 or 4 or 6 or 8 or 10 or 12 or 14 or 16 or 18 or 20 or 22 or 24 or 26 or 29 or 31 or 33 or 35 or 37 or 39 or 41 or 43 or 46 or 50 |  |
| --- | --- |

| 52. internet.mp. or exp Internet/ |  |
| --- | --- |

| 53. limit 52 to (english language and humans) |  |
| --- | --- |

| 54. telemedicine.mp. or exp Telemedicine/ |  |
| --- | --- |

| 55. limit 54 to (english language and humans) |  |
| --- | --- |

| 56. mhealth.mp. |  |
| --- | --- |

| 57. limit 56 to (english language and humans) |  |
| --- | --- |

| 58. ehealth.mp. |  |
| --- | --- |

| 59. limit 58 to (english language and humans) |  |
| --- | --- |

| 60. exp Software/ or web based.mp. |  |
| --- | --- |

| 61. limit 60 to (english language and humans) |  |
| --- | --- |

| 62. website*.mp. |  |
| --- | --- |

| 63. limit 62 to (english language and humans) |  |
| --- | --- |

| 64. exp Cell Phones/ or cell phone*.mp. |  |
| --- | --- |

| 65. limit 64 to (english language and humans) |  |
| --- | --- |

| 66. mobile phone*.mp. |  |
| --- | --- |

| 67. limit 66 to (english language and humans) |  |
| --- | --- |

| 68. smartphone*.mp. or exp Smartphone/ or exp Computers, Handheld/ |  |
| --- | --- |

| 69. limit 68 to (english language and humans) |  |
| --- | --- |

| 70. exp Mobile Applications/ or mobile app*.mp. |  |
| --- | --- |

| 71. limit 70 to (english language and humans) |  |
| --- | --- |

| 72. computer*.mp. |  |
| --- | --- |

| 73. limit 72 to (english language and humans) |  |
| --- | --- |

| 74. exp User-Computer Interface/ |  |
| --- | --- |

| 75. limit 74 to (english language and humans) |  |
| --- | --- |

| 76. mobile health.mp. |  |
| --- | --- |

| 77. limit 76 to (english language and humans) |  |
| --- | --- |

| 78. SMS.mp. |  |
| --- | --- |

| 79. limit 78 to (english language and humans) |  |
| --- | --- |

| 80. exp Electronic Mail/ or exp Text Messaging/ or text messag*.mp. |  |
| --- | --- |

| 81. limit 80 to (english language and humans) |  |
| --- | --- |

| 82. email.mp. |  |
| --- | --- |

| 83. limit 82 to (english language and humans) |  |
| --- | --- |

| 84. web portal.mp. |  |
| --- | --- |

| 85. limit 84 to (english language and humans) |  |
| --- | --- |

| 86. exp "Delivery of Health Care"/ |  |
| --- | --- |

| 87. limit 86 to (english language and humans) |  |
| --- | --- |

| 88. exp Technology/ |  |
| --- | --- |

| 89. limit 88 to (english language and humans) |  |
| --- | --- |

| 90. exp Medical Informatics/ |  |
| --- | --- |

| 91. limit 90 to (english language and humans) |  |
| --- | --- |

| 92. exp Web Browser/ |  |
| --- | --- |

| 93. limit 92 to (english language and humans) |  |
| --- | --- |

| 94. web app*.mp. |  |
| --- | --- |

| 95. limit 94 to (english language and humans) |  |
| --- | --- |

| 96. 53 or 55 or 57 or 59 or 61 or 63 or 65 or 67 or 69 or 71 or 73 or 75 or 77 or 79 or 81 or 83 or 85 or 87 or 89 or 91 or 93 |  |
| --- | --- |

| 97. cancer surviv*.mp. |  |
| --- | --- |

| 98. limit 97 to (english language and humans) |  |
| --- | --- |

| 99. cancer patient*.mp. |  |
| --- | --- |

| 100. limit 99 to (english language and humans) |  |
| --- | --- |

| 101. 98 or 100 |  |
| --- | --- |

| 102. 51 and 96 and 101 |
| --- |

EMBASE (Ovid)

| 1. physical activity.mp. or exp exercise/ or exp physical activity/ |  |
| --- | --- |

| 2. limit 1 to (human and english language) |  |
| --- | --- |

| 3. physical fitness.mp. or exp fitness/ |  |
| --- | --- |

| 4. limit 3 to (human and english language) |  |
| --- | --- |

| 5. fitness.mp. |  |
| --- | --- |

| 6. limit 5 to (human and english language) |  |
| --- | --- |

| 7. exercise*.mp. |  |
| --- | --- |

| 8. limit 7 to (human and english language) |  |
| --- | --- |

| 9. exercise therap*.mp. or exp kinesiotherapy/ |  |
| --- | --- |

| 10. limit 9 to (human and english language) |  |
| --- | --- |

| 11. exp diet/ or exp low calory diet/ or exp low carbohydrate diet/ or diet*.mp. or exp low fat diet/ |  |
| --- | --- |

| 12. limit 11 to (human and english language) |  |
| --- | --- |

| 13. exp dietary intake/ or dietary quality.mp. or exp caloric intake/ |  |
| --- | --- |

| 14. limit 13 to (human and english language) |  |
| --- | --- |

| 15. exp feeding behavior/ |  |
| --- | --- |

| 16. limit 15 to (human and english language) |  |
| --- | --- |

| 17. exp sedentary lifestyle/ or sedentary lifestyle*.mp. |  |
| --- | --- |

| 18. limit 17 to (human and english language) |  |
| --- | --- |

| 19. exp health behavior/ or sedentary behavio?r*.mp. |  |
| --- | --- |

| 20. limit 19 to (human and english language) |  |
| --- | --- |

| 21. exp sitting/ or sedentary time.mp. |  |
| --- | --- |

| 22. limit 21 to (human and english language) |  |
| --- | --- |

| 23. lifestyle.mp. or exp lifestyle modification/ |  |
| --- | --- |

| 24. limit 23 to (human and english language) |  |
| --- | --- |

| 25. health behavio?r*.mp. |  |
| --- | --- |

| 26. limit 25 to (human and english language) |  |
| --- | --- |

| 27. exp behavior change/ or exp health promotion/ or behavio?r change*.mp. |  |
| --- | --- |

| 28. limit 27 to (human and english language) |  |
| --- | --- |

| 29. intervention.mp. or exp intervention study/ |  |
| --- | --- |

| 30. limit 29 to (human and english language) |  |
| --- | --- |

| 31. weight reduction.mp. or exp weight reduction/ |  |
| --- | --- |

| 32. limit 31 to (human and english language) |  |
| --- | --- |

| 33. weight change.mp. or exp weight change/ |  |
| --- | --- |

| 34. limit 33 to (human and english language) |  |
| --- | --- |

| 35. exp weight control/ or weight control.mp. |  |
| --- | --- |

| 36. limit 35 to (human and english language) |  |
| --- | --- |

| 37. sitting.mp. |  |
| --- | --- |

| 38. limit 37 to (human and english language) |  |
| --- | --- |

| 39. body weight.mp. |  |
| --- | --- |

| 40. limit 39 to (human and english language) |  |
| --- | --- |

| 41. weight loss.mp. |  |
| --- | --- |

| 42. limit 41 to (human and english language) |  |
| --- | --- |

| 43. 2 or 4 or 6 or 8 or 10 or 12 or 14 or 16 or 18 or 20 or 22 or 24 or 26 or 28 or 30 or 32 or 34 or 36 or 38 or 40 or 42 |  |
| --- | --- |

| 44. internet.mp. or exp Internet/ |  |
| --- | --- |

| 45. limit 44 to (human and english language) |  |
| --- | --- |

| 46. telemedicine.mp. |  |
| --- | --- |

| 47. limit 46 to (human and english language) |  |
| --- | --- |

| 48. exp medical informatics/ or exp mobile phone/ or exp computer program/ or mhealth.mp. |  |
| --- | --- |

| 49. limit 48 to (human and english language) |  |
| --- | --- |

| 50. ehealth.mp. or exp telehealth/ |  |
| --- | --- |

| 51. limit 50 to (human and english language) |  |
| --- | --- |

| 52. telehealth.mp. |  |
| --- | --- |

| 53. limit 52 to (human and english language) |  |
| --- | --- |

| 54. web based.mp. |  |
| --- | --- |

| 55. limit 54 to (human and english language) |  |
| --- | --- |

| 56. exp online system/ or website*.mp. |  |
| --- | --- |

| 57. limit 56 to (human and english language) |  |
| --- | --- |

| 58. cell phone*.mp. or exp text messaging/ |  |
| --- | --- |

| 59. limit 58 to (human and english language) |  |
| --- | --- |

| 60. mobile phone*.mp. |  |
| --- | --- |

| 61. limit 60 to (human and english language) |  |
| --- | --- |

| 62. exp personal digital assistant/ or smartphone*.mp. |  |
| --- | --- |

| 63. limit 62 to (human and english language) |  |
| --- | --- |

| 64. exp mobile application/ or mobile app*.mp. |  |
| --- | --- |

| 65. limit 64 to (human and english language) |  |
| --- | --- |

| 66. exp teleconsultation/ |  |
| --- | --- |

| 67. limit 66 to (human and english language) |  |
| --- | --- |

| 68. exp telemonitoring/ |  |
| --- | --- |

| 69. limit 68 to (human and english language) |  |
| --- | --- |

| 70. exp teletherapy/ |  |
| --- | --- |

| 71. limit 70 to (human and english language) |  |
| --- | --- |

| 72. email.mp. or exp e-mail/ |  |
| --- | --- |

| 73. limit 72 to (human and english language) |  |
| --- | --- |

| 74. mobile health.mp. |  |
| --- | --- |

| 75. limit 74 to (human and english language) |  |
| --- | --- |

| 76. SMS.mp. |  |
| --- | --- |

| 77. limit 76 to (human and english language) |  |
| --- | --- |

| 78. text messag*.mp. |  |
| --- | --- |

| 79. limit 78 to (human and english language) |  |
| --- | --- |

| 80. mobile device*.mp. |  |
| --- | --- |

| 81. limit 80 to (human and english language) |  |
| --- | --- |

| 82. 45 or 47 or 49 or 51 or 53 or 55 or 57 or 59 or 61 or 63 or 65 or 67 or 69 or 71 or 73 or 75 or 77 or 79 or 81 |  |
| --- | --- |

| 83. exp cancer survivor/ or cancer survivor*.mp. |  |
| --- | --- |

| 84. limit 83 to (human and english language) |  |
| --- | --- |

| 85. exp cancer patient/ or cancer patient*.mp. |  |
| --- | --- |

| 86. limit 85 to (human and english language) |  |
| --- | --- |

| 87. 84 or 86 |  |
| --- | --- |

| 88. 43 and 82 and 87 |
| --- |

PsycINFO (Ovid)

| 1. physical activity.mp. or exp Physical Activity/ |
| --- |

| 2. limit 1 to (human and english language) |  |
| --- | --- |

| 3. physical fitness.mp. or exp Physical Fitness/ |  |
| --- | --- |

| 4. limit 3 to (human and english language) |  |
| --- | --- |

| 5. exp Exercise/ or exp Physical Education/ or exp Health Promotion/ or exp Health/ or fitness.mp. |
| --- |

| 6. limit 5 to (human and english language) |  |
| --- | --- |

| 7. exercise*.mp. |  |
| --- | --- |

| 8. limit 7 to (human and english language) |  |
| --- | --- |

| 9. exercise therap*.mp. [mp=title, abstract, heading word, table of contents, key concepts, original title, tests & measures] |  |
| --- | --- |

| 10. limit 9 to (human and english language) |  |
| --- | --- |

| 11. exp Eating Behavior/ or exp Food Intake/ or exp Diets/ or exp Food/ or diet*.mp. |  |
| --- | --- |

| 12. limit 11 to (human and english language) |  |
| --- | --- |

| 13. exp Body Weight/ or dietary quality.mp. or exp Health Behavior/ or exp Weight Control/ |  |
| --- | --- |

| 14. limit 13 to (human and english language) |  |
| --- | --- |

| 15. exp Lifestyle/ or exp Activity Level/ or sedentary lifestyle*.mp. |  |
| --- | --- |

| 16. limit 15 to (human and english language) |  |
| --- | --- |

| 17. sedentary behavio?r*.mp. |  |
| --- | --- |

| 18. limit 17 to (human and english language) |  |
| --- | --- |

| 19. sedentary time.mp. |  |
| --- | --- |

| 20. limit 19 to (human and english language) |  |
| --- | --- |

| 21. exp Lifestyle Changes/ or lifestyle.mp. |  |
| --- | --- |

| 22. limit 21 to (human and english language) |  |
| --- | --- |

| 23. health behavio?r*.mp. |  |
| --- | --- |

| 24. limit 23 to (human and english language) |  |
| --- | --- |

| 25. exp Behavior Change/ or exp Intervention/ or behavio?r change*.mp. |  |
| --- | --- |

| 26. limit 25 to (human and english language) |  |
| --- | --- |

| 27. self management.mp. or exp Self Management/ |  |
| --- | --- |

| 28. limit 27 to (human and english language) |  |
| --- | --- |

| 29. intervention.mp. [mp=title, abstract, heading word, table of contents, key concepts, original title, tests & measures] |  |
| --- | --- |

| 30. limit 29 to (human and english language) |  |
| --- | --- |

| 31. sitting.mp. [mp=title, abstract, heading word, table of contents, key concepts, original title, tests & measures] |  |
| --- | --- |

| 32. limit 31 to (human and english language) |  |
| --- | --- |

| 33. weight control.mp. [mp=title, abstract, heading word, table of contents, key concepts, original title, tests & measures] |  |
| --- | --- |

| 34. limit 33 to (human and english language) |  |
| --- | --- |

| 35. body weight.mp. [mp=title, abstract, heading word, table of contents, key concepts, original title, tests & measures] |  |
| --- | --- |

| 36. limit 35 to (human and english language) |  |
| --- | --- |

| 37. weight reduction.mp. [mp=title, abstract, heading word, table of contents, key concepts, original title, tests & measures] |  |
| --- | --- |

| 38. limit 37 to (human and english language) |  |
| --- | --- |

| 39. weight loss.mp. [mp=title, abstract, heading word, table of contents, key concepts, original title, tests & measures] |  |
| --- | --- |

| 40. limit 39 to (human and english language) |  |
| --- | --- |

| 41. 2 or 4 or 6 or 8 or 10 or 12 or 14 or 16 or 18 or 20 or 22 or 24 or 26 or 28 or 30 or 32 or 34 or 36 or 38 or 40 |  |
| --- | --- |

| 42. internet.mp. or exp Internet/ |  |
| --- | --- |

| 43. limit 42 to (human and english language) |  |
| --- | --- |

| 44. telemedicine.mp. or exp Telemedicine/ |  |
| --- | --- |

| 45. limit 44 to (human and english language) |  |
| --- | --- |

| 46. exp Mobile Devices/ or exp Cellular Phones/ or exp Technology/ or mhealth.mp. or exp Health Care Delivery/ |  |
| --- | --- |

| 47. limit 46 to (human and english language) |  |
| --- | --- |

| 48. exp Electronic Communication/ or ehealth.mp. |  |
| --- | --- |

| 49. limit 48 to (human and english language) |  |
| --- | --- |

| 50. exp Computer Mediated Communication/ or exp Websites/ or web based.mp. or exp Online Therapy/ |  |
| --- | --- |

| 51. limit 50 to (human and english language) |  |
| --- | --- |

| 52. exp Computer Applications/ or website*.mp. |  |
| --- | --- |

| 53. limit 52 to (human and english language) |  |
| --- | --- |

| 54. cell-phone*.mp. |  |
| --- | --- |

| 55. limit 54 to (human and english language) |  |
| --- | --- |

| 56. mobile phone*.mp. |  |
| --- | --- |

| 57. limit 56 to (human and english language) |  |
| --- | --- |

| 58. smartphone*.mp. |  |
| --- | --- |

| 59. limit 58 to (human and english language) |  |
| --- | --- |

| 60. mobile app*.mp. |  |
| --- | --- |

| 61. limit 60 to (human and english language) |  |
| --- | --- |

| 62. computer*.mp. |  |
| --- | --- |

| 63. limit 62 to (human and english language) |  |
| --- | --- |

| 64. mobile health.mp. |  |
| --- | --- |

| 65. limit 64 to (human and english language) |  |
| --- | --- |

| 66. exp Messages/ or SMS.mp. |  |
| --- | --- |

| 67. limit 66 to (human and english language) |  |
| --- | --- |

| 68. email.mp. |  |
| --- | --- |

| 69. limit 68 to (human and english language) |  |
| --- | --- |

| 70. exp Information Technology/ or web portal.mp. |  |
| --- | --- |

| 71. limit 70 to (human and english language) |  |
| --- | --- |

| 72. exp Computers/ |  |
| --- | --- |

| 73. limit 72 to (human and english language) |  |
| --- | --- |

| 74. exp Information Systems/ |  |
| --- | --- |

| 75. limit 74 to (human and english language) |  |
| --- | --- |

| 76. text messag*.mp. [mp=title, abstract, heading word, table of contents, key concepts, original title, tests & measures] |  |
| --- | --- |

| 77. limit 76 to (human and english language) |  |
| --- | --- |

| 78. 43 or 45 or 47 or 49 or 51 or 53 or 55 or 57 or 59 or 61 or 63 or 65 or 67 or 69 or 71 or 73 or 75 or 77 |  |
| --- | --- |

| 79. cancer surviv*.mp. [mp=title, abstract, heading word, table of contents, key concepts, original title, tests & measures] |  |
| --- | --- |

| 80. limit 79 to (human and english language) |  |
| --- | --- |

| 81. cancer patient*.mp. [mp=title, abstract, heading word, table of contents, key concepts, original title, tests & measures] |  |
| --- | --- |

| 82. limit 81 to (human and english language) |  |
| --- | --- |

| 83. exp Survivors/ |  |
| --- | --- |

| 84. limit 83 to (human and english language) |  |
| --- | --- |

| 85. 80 or 82 or 84 |  |
| --- | --- |

| 86. 41 and 78 and 85 |
| --- |

CINAHL (EBSCOhost)

| S90 | S62 AND S86 AND S89 |
| --- | --- |
| S89 | S87 OR S88 |
| S88 | cancer patient* |
| S87 | (MH "Cancer Survivors") OR "cancer surviv*" OR (MH "Cancer Patients") |
| S86 | S63 OR S64 OR S65 OR S66 OR S67 OR S68 OR S69 OR S70 OR S71 OR S72 OR S73 OR S74 OR S75 OR S76 OR S77 OR S78 OR S79 OR S80 OR S81 OR S82 OR S83 OR S84 OR S85 |
| S85 | (MH "Medical Informatics") |
| S84 | (MH "Health Informatics+") |
| S83 | (MH "Technology+") |
| S82 | (MH "Health Care Delivery+") |
| S81 | (MH "Instant Messaging") |
| S80 | "email" |
| S79 | SMS |
| S78 | "mobile health" |
| S77 | (MH "Text Messaging") OR "text messag*" |
| S76 | (MH "Computers, Hand-Held+") |
| S75 | "computer*" |
| S74 | (MH "Mobile Applications") OR "mobile app*" |
| S73 | "smartphone*" |
| S72 | mobile phone* |
| S71 | (MH "Cellular Phone+") OR (MH "Smartphone+") OR "cell phone*" |
| S70 | "web 2.0" |
| S69 | "website*" |
| S68 | "web portal" OR (MH "Web Browsers") |
| S67 | "web based" |
| S66 | "ehealth" |
| S65 | "mhealth" |
| S64 | (MH "Telemedicine+") OR "telemedicine" OR (MH "Telehealth+") |
| S63 | (MH "Internet+") OR "internet" |
| S62 | S2 OR S4 OR S6 OR S8 OR S10 OR S12 OR S14 OR S16 OR S18 OR S20 OR S22 OR S24 OR S26 OR S28 OR S29 OR S31 OR S33 OR S35 OR S37 OR S39 OR S41 OR S43 OR S45 OR S47 OR S49 OR S51 OR S53 OR S55 OR S57 OR S59 OR S61 |
| S61 | (MH "Health+") |
| S60 | (MH "Health+") |
| S59 | (MH "Health Promotion+") |
| S58 | (MH "Health Promotion+") |
| S57 | (MH "Behavior Modification+") |
| S56 | (MH "Behavior Modification+") |
| S55 | (MH "Eating Behavior+") |
| S54 | (MH "Eating Behavior+") |
| S53 | (MH "Food Intake+") OR (MH "Energy Intake") |
| S52 | (MH "Food Intake+") OR (MH "Energy Intake") |
| S51 | (MH "Diet Therapy+") |
| S50 | (MH "Diet Therapy+") |
| S49 | (MH "Education, Physical Therapy") OR (MH "Physical Education and Training+") OR (MH "Physical Therapists") |
| S48 | (MH "Education, Physical Therapy") OR (MH "Physical Education and Training+") OR (MH "Physical Therapists") |
| S47 | weight loss |
| S46 | weight loss |
| S45 | weight reduction |
| S44 | weight reduction |
| S43 | body weight |
| S42 | body weight |
| S41 | (MH "Weight Control") OR "weight control" OR (MH "Body Weight Changes+") |
| S40 | (MH "Weight Control") OR "weight control" OR (MH "Body Weight Changes+") |
| S39 | (MH "Sitting") OR "sitting" |
| S38 | (MH "Sitting") OR "sitting" |
| S37 | "intervention" OR (MH "Intervention Trials") |
| S36 | "intervention" OR (MH "Intervention Trials") |
| S35 | (MH "Self Care+") OR "self management" |
| S34 | (MH "Self Care+") OR "self management" |
| S33 | behavior change* |
| S32 | behavior change* |
| S31 | (MH "Behavioral Changes") OR "behaviour change*" |
| S30 | (MH "Behavioral Changes") OR "behaviour change" |
| S29 | health behaviour |
| S28 | (MH "Health Behavior+") OR "health behavior*" |
| S27 | (MH "Health Behavior+") OR "health behavior*" |
| S26 | (MH "Life Style Changes") OR "lifestyle" |
| S25 | (MH "Life Style Changes") OR "lifestyle" |
| S24 | sedentary time |
| S23 | sedentary time |
| S22 | sedentary behavior |
| S21 | sedentary behavior |
| S20 | "sedentary behaviour" |
| S19 | "sedentary behaviour" |
| S18 | (MH "Life Style, Sedentary") OR "sedentary lifestyle*" |
| S17 | (MH "Life Style, Sedentary") OR "sedentary lifestyle*" |
| S16 | dietary quality |
| S15 | dietary quality |
| S14 | (MH "Diet, Reducing") OR (MH "Diet+") |
| S13 | (MH "Diet, Reducing") OR (MH "Diet+") |
| S12 | "diet*" |
| S11 | "diet*" |
| S10 | exercise therap* |
| S9 | exercise therap* |
| S8 | exercise* |
| S7 | exercise* |
| S6 | "fitness" |
| S5 | "fitness" |
| S4 | (MH "Physical Fitness+") OR "physical fitness" OR (MH "Exercise+") |
| S3 | (MH "Physical Fitness+") OR "physical fitness" OR (MH "Exercise+") |
| S2 | (MH "Physical Activity") OR "physical activity" OR (MH "Physical Fitness+") |
| S1 | (MH "Physical Activity") OR "physical activity" OR (MH "Physical Fitness+") |
