# Supplementary material for: Digital health behaviour change interventions targeting physical activity and diet in cancer survivors: a systematic review and meta-analysis
Source: J Cancer Surviv. 2017 Aug 4;11(6):704–19. doi: 10.1007/s11764-017-0632-1 (PMC5671545; doi:10.1007/s11764-017-0632-1)
Supplement: Supplementary file 2 — (DOCX 51 kb) [file 11764_2017_632_MOESM2_ESM.docx]

Article title: Digital health behaviour change interventions targeting physical activity and diet in cancer survivors: a systematic review and meta-analysis

Journal: Journal of Cancer Survivorship

Author names: Anna L Roberts, Abigail Fisher, Lee Smith, Malgorzata Heinrich, Henry W W Potts

Corresponding author: Abigail Fisher, Department of Behavioural Science & Health, University College London, Gower Street, London, WC1E 6BT, United Kingdom.

Email: [abigail.fisher@ucl.ac.uk](mailto:abigail.fisher@ucl.ac.uk)


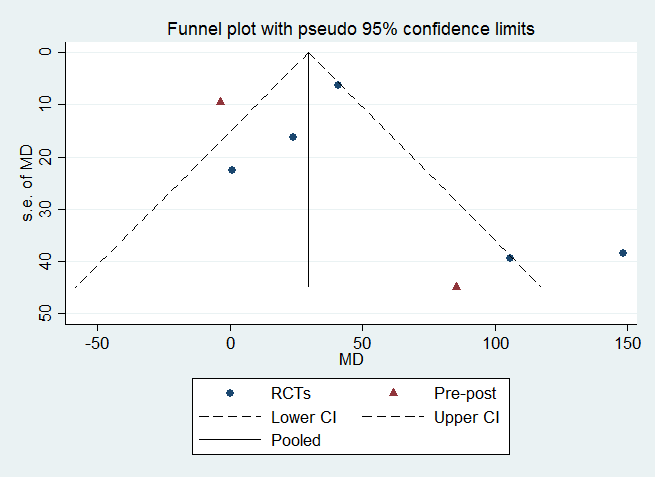


Figure 1: Funnel plot illustrating possible publication bias for studies assessing MVPA

Figure 2: Meta-analysis of DBCIs on BMI/weight measurements.

N.B. p-values shown in Figure 2 represent significance for testing of heterogeneity

Figure 3: Meta-analysis of DBCIs on fatigue.

N.B. p-values shown in Figure 3 represent significance for testing of heterogeneity

Figure 4: Meta-analysis of DBCIs on cancer-specific QoL.

N.B. p-values shown in Figure 4 represent significance for testing of heterogeneity
